# Cadmium and Lead Levels in Blood and Arsenic Levels in Urine among Schoolchildren Living in Contaminated Glassworks Areas, Sweden

**DOI:** 10.3390/ijerph17207382

**Published:** 2020-10-10

**Authors:** Kristoffer Mattisson, Eva Tekavec, Thomas Lundh, Emilie Stroh

**Affiliations:** Division of Occupational and Environmental Medicine, Lund University, 22362 Lund, Sweden; eva.tekavec@med.lu.se (E.T.); thomas.lundh@med.lu.se (T.L.); emilie.stroh@med.lu.se (E.S.)

**Keywords:** biological monitoring, lead, cadmium, arsenic, children

## Abstract

The Kingdom of Crystal, an area in southern Sweden famous for its many glassworks, is historically heavily burdened by pollution from this industry. Glass crust containing cadmium (Cd), lead (Pb), and arsenic (As) has been deposited around the area and used as filling. The purpose of this study was to monitor whether the high levels of metals in the contaminated soil were reflected in blood and urine among school children in this area. Blood and urine samples were collected from 87 children in 2017. The levels of cadmium (Cd-B) and lead (Pb-B) found in blood were determined by inductively coupled plasma mass spectrometry (ICP-MS). The speciation of As in urine (As-U) was performed by ion chromatography. The geometric mean of Cd-B and Pb-B among the children were 0.09 μg/L and 9.9 μg/L respectively. The geometric mean of inorganic As (AsIII and AsV) with metabolites in urine was 6.1 μg/L and 6.94 μg/g creatinine. Children in the study area had blood levels of Pb and Cd that correspond to levels generally found in Swedish children. The levels of inorganic As and its metabolites in urine were low and in the same magnitude as other children in Europe and the U.S.

## 1. Introduction

The area called “The Kingdom of Crystal” in southern Sweden is famous for its many glasswork industries. The majority of these industries are no longer in use today, but the historical reputation of artisanship makes this area a well-visited tourist attraction. Unfortunately, the area is highly contaminated with heavy metals previously used in glass production. For instance, lead (Pb) as a stabilizer, arsenic (As) as fining agent and cadmium (Cd) as pigment [1]. Waste materials from the glass factories, called glassworks, often became filling material for construction purposes and were spread across nearby villages. Many buildings here, including residences and schools, were constructed after the establishment of the glassworks. Consequently, high levels of Cd, Pb and As were deposited in the soil [2] in places where people can be expected to stay on a daily basis. A prior study compiled the deposition of these three pollutants in the soil at 22 glassworks properties considered high-risk, scattered around the Kingdom of Cristal [1]. In the report, 130,000 m^2^ of glass were estimated to have been deposited around these 22 glassworks, which contained 19 ton Cd, 1600 ton Pb and 310 ton As. This resulted in mean levels in the soil just above 10 mg/kg dw for Cd, 1000 mg/kg dw for Pb and 200 mg/kg dw for As. The mean level at landfills was 10 times higher for Cd and As and 6 times higher for Pb compared to usable land. Exposure to these chemicals could pose a risk for acute and chronic adverse health effects among those living nearby. 

The most important routes of exposure to Cd are through the consumption of food, and among adults, also smoking [3,4]. Uptake of ingested Cd in the gastrointestinal tract varies from 2–20%, and having low iron status can increase one’s uptake [5,6]. Cd accumulates in the kidneys and has a long half-life in the body, up to several decades [3]. The concentration of Cd in the blood therefore reflects both current exposure and the total body burden. Long-term Cd exposure is a risk factor for kidney damage and osteoporosis [7,8,9,10]. The European Food Authority, EFSA, reckons that the margins to adverse health manifestations are subtle or erased, thus further limitation of exposures are warranted [4]. Moreover, Cd is classified as carcinogenic to humans (IARC 2012, group 1) [11]. Cd exposure has also been associated with increased risk of stroke and cardiovascular events [12], as well as impact on male fertility and impaired fetal growth due to decreased placental circulation [3].

The main exposure route for Pb today is through food. Many foods contain low levels of Pb, but some products, such as shellfish and game meat from animals shot with Pb shotguns, can contain higher levels. In the past, tetraethyl Pb was used as an octane-raising and anti-knock additive in gasoline, which was a significant source of Pb exposure through contaminated air and soil. Pb is bound to 99% to hemoglobin in the red blood cells and has a half-life of one to two months in the blood, while the half-life of Pb from the skeletal pool can span several decades [3]. Uptake via the gastrointestinal tract is higher among children (40–50%), compared to adults (3–10%) and is dependent upon factors such as nutritional status and physiological characteristics of Pb in the medium ingested [13]. The developing brain is the critical organ of Pb exposure. A decrease in IQ scores has been detected in large population-based studies at a Pb-B level of 12 μg/L [14,15,16]. Pregnant women, and their developing fetuses, as well as children, have been found to have a Pb exposure at, and even slightly above, the reference value of 0.5 μg Pb/kg body burden [17]. This corresponds to a blood Pb concentration of 12 μg/L. Since no lower limit threshold value has been identified, Pb exposure should be kept as low as possible, and all additional exposure is considered unwanted. Acute intoxication in children can cause toxic encephalopathy, a severe condition, which requires medical emergency treatment [18]. International Agency for Research on Cancer, IARC, has classified inorganic Pb compounds as probably carcinogenic (Group 2A; IARC 2006) [19]. Pb exposure can cause reproductive disorder for both men and women [3].

Arsenic exposure generally arises from ingestion of food and drinking water [20]. With this pollutant occurring naturally in bedrock and groundwater, some crops, such as rice, may absorb inorganic As from soil contaminants and water [20]. In addition to As occurring naturally, it has also been used for wood impregnation, alloy production, preservation of taxidermy animals and, in some countries, for pesticide use [21]. Organic As compounds (Arsenobetain, Arsenocholine) can be found in fish and seafood and intake of seafood and fish can cause the levels of organic As to rise sharply in the urine [22,23,24,25]. Arsenic is a metalloid that can cause both acute and chronic health impacts [26], but its toxicity depends on the form of As, the mode of uptake and individual sensitivity [22,23,25]. Organic As is significantly less toxic than inorganic. Uptake from contaminated soil through ingestion varies considerably (3–75%) [27]. Some uptake also occurs through inhalation and skin contact [28,29]. Since the half-life of inorganic As in the urine is short, most of it is metabolized within a few days. Still, several studies have demonstrated an increased risk of cardiovascular disease and diabetes following exposure to high levels of inorganic As [30,31]. Additionally, exposure during fetal development has been linked to an increased risk of adverse neurological effects and an impaired immune system [32,33]. As has been observed in studies concerning populations highly exposed to inorganic As through drinking water, such as in Bangladesh and Taiwan, chronic As exposure can cause hyperkeratosis of the palms and soles of the feet, hypo/hyper pigmentation of the skin and, vascular affection [3]. Furthermore, As is carcinogenic (IARC class 1) and associated with excess risk of cancer of the lungs, skin and urinary bladder [21].

Given the high levels of Cd, Pb and As in soil recorded around the glassworks sites [1], such adverse health outcomes may be a threat to the surrounding population. A potential exposure route for those residing in the study area is the consumption of contaminated food. A recent study from The Kingdom of Cristal [34] analyzed the levels of Cd, Pb and As in a range of vegetables grown and collected near 22 contaminated glasswork sites. Their results indicated that only levels of Pb were higher in vegetables from glasswork sites compared to those grown and harvested in reference areas. Even so, estimated exposure assessments showed significantly higher exposure for low, medium and high consumers of vegetables from the contaminated areas. Intake of vegetables alone did not result in exposure levels exceeding the tolerable daily intake, but there are also other potentially important sources (such as locally caught fresh water fish) and exposure routes, e.g., inhalation dust or ingestion of soil, that can contribute to health hazardous exposures. 

While this study included only estimated exposure assessments for adults, children are especially sensitive to these metals for several reasons. As their bodies are still developing, for instance, children’s metabolic systems are more immature than adults’ [35]. A child ingesting or inhaling the same amount result in a larger internal dose due to the child’s lower weight [35]. Finally, children’s behavior, including spending more time spent outdoors and having less sanitary hand-to-mouth habits, differs from that of adults [35]. The general levels of B-Pb among children have previously been rather well monitored, but less is known about B-Cd [36,37]. Prior studies have also shown an association between living in a contaminated area and having higher levels of B-Pb [38,39,40]. Similar associations have also been found for children living in contaminated areas and having higher levels of B-Cd and As in urine (U-As) [41]. 

The cost to decontaminate soil is high, and health risk assessments exaggerating the risk of living or spending time in these areas might cause anxiety and negatively affect residents’ wellbeing. It is, therefore, important to understand the exposure levels of people, especially children, living in polluted areas, in order to better estimate the risks and, thereby, ascertain the potential mitigation and prevention measures needed. The purpose of this project was therefore to monitor whether the high levels of pollutants (Cd, Pb and As) in the contaminated soil in this area were reflected in blood and urine among schoolchildren living there. Furthermore, a large recently published study on contaminants in blood and urine among adolescents (grade 5, 8 and 11) in Sweden, collected in 2016–2017 by the Swedish Environmental Protection Agency (EPA), will make it possible to compare our results in this study with general levels in Sweden [42], as well as previous studies of biomonitoring of these pollutants in children in southern Sweden [37,43,44] and a historical study on children from the area [45].

## 2. Materials and Methods 

### 2.1. Study Area

**Figure 1:**
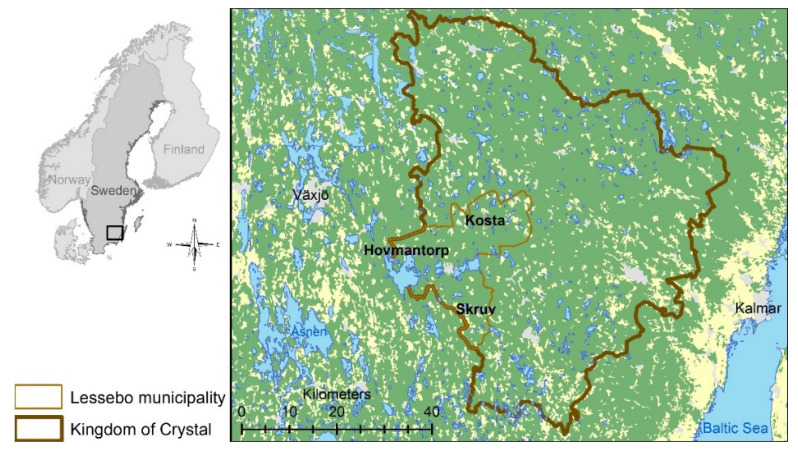
Shows the study area Lessebo municipality and its location in the Kingdom of Crystal. The area’s location in Sweden is presented to the left in the figure.

The study sample was selected from three towns in the area of “The Kingdom of Crystals”; Kosta, Hovmantorp and Skruv (Figure 1). All of these situated in Lessebo municipality in southern Sweden. Lessebo municipality has a tradition of glass production dating back to the beginning of the 17th century [46]. The area is sparsely populated, with 21 people per m^2^ in the municipality. Hovmantorp has just above 3000 inhabitants, Kosta and Skruv below 1000. A site-specific compilation of levels in the soil around seven glassworks in Lessebo municipality, based on 291 historical measurements, showed mean levels of Pb of 1298 mg/kg dw (max 73,500 mg/kg dw), mean levels of Cd of 5 mg/kg dw (max 374 mg/kg dw) and mean levels of As 73 mg/kg dw (Max 3530 mg/kg dw) [47]. 

### 2.2. Study Sample

All school children in grade 2–4 (equivalent to 8–10 years of age) in Kosta, Hovmantorp and Skruv in Lessebo municipality were invited to participate in the study (N = 237). Of these, 87 participated (37%), of which 85 left both blood and urine samples and two only urine samples. The study was approved by Lund Ethical review board on the 29 June 2017 (dnr 2017/182), as biological samples from children were to be collected. Before taking samples, interview questions (File S1) and consent forms (File S2) were sent to the parents to fill in with the children. The questionnaire contained questions regarding other risk factors and exposure routes for Cd, Pb and As. For instance, being born and raised in the area impacted the time period spent in the area where exposure for the contaminated soil could take place. Drinking water from own well and eating locally caught fish or locally grown vegetables could lead to increased oral exposure of contaminants from the area. Having smoking parents could increase the exposure of Cd via cigarette smoke and playing with tin soldiers increase exposure to Pb. Participation in the study could be terminated at any time. Contact was also established with principals and teachers at the schools and communication officers at the municipality and information about the study spread to the public through a press release. Blood and urine sampling were carried out within a week in the autumn of 2017; each individual child left samples at one occasion. The sample collection was performed in the period about 1.5 month after the children had been back in school after summer vacation, in order for the exposure at home to have stabilized. Blood was retrieved from a cubital vein into heparinized evacuated tubes (Vacuette; Greiner-BioOne, GmbH, Frickhausen, Germany) and urine samples were collected in acid-washed paper cups and transferred to acid-washed 13 mL polypropene tubes (Sarstedt, Nümbrecht, Germany). Blood and urine samples were kept refrigerated during the sampling period and during the transport to the laboratory, where they were stored frozen until analyzed.

### 2.3. Analysis of Blood Samples

The blood samples were thawed and the levels of B-Pb and B-Cd in the samples were determined by inductively coupled plasma mass spectrometry (ICP-MS; iCAP Q, Thermo Fisher Scientific, GmbH, Bremen, Germany), equipped with collision cell with kinetic discrimination (KED) and helium as collision gas. The detection limit was for B-Pb 0.05 µg/L and B-Cd 0.02 µg/L. External reference material from Seronorm, (Sero AS, Billingstad, Norway) and from G-EQUAS (The German External Quality Assessment Scheme Erlangen Germany) was analyzed in the same analysis round as the current blood samples. The results for Seronorm (Lot 1406263) were for B-Pb 11 ± 0.15 µg/L (mean ± SD) vs. recommended content of 7.9–12 µg/L for B-Cd 0.23 ± 0.01 µg/L vs. 0.17–0.40 µg/L). For G-EQUAS (R59 7B), B-Pb 30 ± 0.44 µg/L vs. recommended content of 24–34 µg/L for B-Cd 0.78 ± 0.02 µg/L vs. 0.62–1.0 µg/L.

### 2.4. Analysis of Urine 

The urine samples were thawed and the speciation of As in urine samples was performed by ion chromatography (Dionex 5000+, Thermo Fisher Scientific, GmbH, Bremen, Germany) linked to ICP-MS (iQAP Q, KED mode). Detection limit of the As metabolites was for Arsenite (AsIII) 0.25 µg/L, Arsenate (AsV) 0.30 µg/L, Dimethylarsin acid (DMA) 0.25 µg/L and Monomethylarson acid (MMA) 0.25 µg/L L. External reference material from the German External Quality Assessment Scheme (G-EQUAS), Erlangen Germany (R61 8B) was used in the analyses. The results for the external controls (n = 9) for DMA were 12 ± 1.2 (mean, SD) vs. recommended 13 (11–16) µg/L, MMA 2.5 ± 0.14 vs. 2.5 (2.0 2.9 µg/L, AsIII 0.97 ± 0.12 vs. 0.88 (0.55–1.2) µg/L and AsV 1.5 ± 0.21 vs. 0.93 (0.51–1.4) µg/L.

### 2.5. Statistical Analysis

Geometrical mean values were calculated for levels of heavy metals in blood and As in urine. Based on different risk factors for exposure and behavior addressed by the questionnaire, the study population were stratified into groups. Independent sample t-tests were performed for each individual group division to compare if the arithmetic mean values differed between the log-transformed values. Levene’s test for equality of variance were used to decide if equal variance should be assumed or not. Pearson’s correlation test were performed to test for correlation between B-Cd and B-Pb. All statistical analyses were performed in IBMs SPSS version 24 (IBM, Armonk, NY, USA).

## 3. Results

In the study population, there was an even distribution between boys and girls, and the majority of the study participants attended Hovmantorp school (Table 1). The distribution was relatively even between the different age groups, with most of the children attending grade 3. More than half of the children were born or raised in the area. For those who did not grow up in the area, the average time spent there was 3.7 years; only a small proportion (6%) had lived in the area for less than 2 years. 

From the questionnaire, it was apparent that only a few children played or casted tin soldiers, fished with Pb sinker or shoot with air rifle (12%). Almost half (46%) of the children ate locally grown vegetables and 22% responded that their residence had water from its own well and about a 25% of the children ate locally caught freshwater fish. A large majority (90 %) claimed that the parents did not smoke tobacco.

### 3.1. Cd and Pb in Blood

The geometric mean (GM) of Cd in the blood of the sampled children was 0.09 µg/L and the maximum value was 0.26 µg/L (Table 2). For Pb in blood, the geometric mean was 9.9 µg/L and the maximum was 42 µg/L (Table 2). There was a significant correlation between the blood levels (Pearson correlation test, 0.374 **).

The GM values in the blood for the children were stratified into different groups (Table 3). The children who were not born/raised in the area had significantly higher levels of both Cd-B (*p*-value: 0.02) and Pb-B (*p*-value: 0.02). 

### 3.2. As in Urine

The GM of inorganic As in urine, e.g., AsIII, AsV, DMA and MMA, were 6.1 µg/L and 6.9 µg/g creatinine. Table 4 also presents geometric mean values unadjusted and creatinine adjusted for DMA, MMA, AsIII and AsV separately.

Independent t-tests were performed for each stratified group to compare if the geometric mean values differed for unadjusted and creatinine-adjusted values, respectively (Table 5). 

## 4. Discussion

Children in the study area had blood levels of Pb and Cd that corresponded to levels found in children in general in Sweden [37,42,43,44]. The levels of inorganic As and its metabolites in urine also did not differ in these children compared to Swedish children and studies performed in children in the rest of Europe [36,42,48,49]. Thus, this study indicates that, children living in the contaminated areas in the Kingdom of Crystal do not appear to be exposed to these metals and metalloid in levels more than the general population. Even so, no detailed information about activity pattern and behavior were known, and it is therefore not possible to certainly know whether the children actually were exposed to the contaminated soil or not. 

### 4.1. Cd

The B-Cd of children in Lessebo (0.09 µg/L) corresponds to the levels measured in the city Landskrona (0.10 µg/L) the same year [43]. The levels were also in the same magnitude as in adolescents in Sweden (0.16 µg/L) [42]. In an analysis of historical exposure of 1120 children in Landskrona and Trelleborg, the geometric mean was 0.10 µg/L, and possibly with a slightly decreasing trend from 0.11 µg/L in 1986 to 0.09 µg/L in 2013 [37]. The children thus seem to have a similar exposure as that of other children in southern Sweden and Swedish adolescents, and can thus not be considered to have elevated levels in the blood. The general B-Cd in children in Sweden are in level with the rest of Europe, but low in an international comparison [36]. 

The stratified analysis showed a statistically significant difference (*p*-value: 0.02) between study participants not born and/or raised in the area B-Cd (0.11 µg/L), compared to those born and/or raised in the area (0.08 µg/L). Even so, none of these groups could be considered to have elevated levels.

B-Cd in the study population are in level with the general population, and there seem to be no increased risks of adverse health effect due to Cd exposure. Even so, since exposure at current levels is close to the effect limits that may over time cause kidney damage and osteoporosis, there is reason to reduce the exposure to the general population [50]. Adverse health effects from Cd exposure, e.g., kidney damage and osteoporosis, manifests at older age. In Sweden, 10% of all fractures are estimated because osteoporosis is due to the public’s Cd exposure, which is estimated to cost society around SEK 4.2 billion per year [51].

### 4.2. Pb

The geometric mean of B-Pb in the study population was 9.9 µg/L. This is less than one third of the levels found in an earlier study in the same area from 1986 (35 µg/L) [45]. Children who grow up in the area today seem to have a significantly lower exposure than their parents. In comparison with a reference population in Landskrona in 2017 (7.8 µg/L), the geometric mean is slightly higher [43]. The levels are also in the same magnitude as in Swedish adolescents (8.5 µg/L) [42].

For the stratified comparison of mean values that were carried out for B-Pb, there was a statistically significant increase in B-Pb for children that were not born or raised in the area, compared to those who were (*p*-value: 0.02). Two children who participated in the study had Pb levels in the blood that were assessed to significantly exceed the average level. These children were followed up with additional samples and a home visit. The additional samples showed decreasing Pb levels in the blood over a six-month period, but the reason for their temporally higher B-Pb levels in the study could not be determined.

The European Food and Safety Authority (EFSA) has set BMDL (benchmark dose lower limit) for Pb to 0.5 µg/kg body weight and day (the level that a child is considered to be exposed to daily throughout their lifetime without suffering any adverse health effects), which correlates to B-Pb of 12 µg/L [52]. Even though levels of Pb and Cd were not elevated in comparison to what is seen in the general population, there is a need to strive for a reduction in exposure. This argument is in alignment with the EFSA recommendation; to omit adverse health effects [52]. For Pb, there is no lower to protect from IQ impairment. For Cd, we are already on the margins, since it is present in our daily food.

### 4.3. As

The geometric mean of inorganic As, including the metabolites DMA and MMA, in the urine was 6.1 µg/L and 6.9 µg As/g Creatinine. Few studies with specification have examined the levels of inorganic As in urine in Sweden. The study on Swedish adolescents was density adjusted, and it was therefore not possible to directly compare the results, but that study showed a mean value for inorganic As, DMA and MMA on 4.1 µg/kg urine [42]. Historically, however, the level at the study site has been measured and the geometric mean value for inorganic As, including DMA and MMA, was 5.1 µg As/g creatinine [45]. Thus, the levels present in the children in this study are slightly higher. The U.S. Public Health Authority (CDC) has compiled figures on the levels of inorganic As (AsIII and AsV) and its metabolites (DMA and MMA) in urine in 380 children in the United States, sampled in 2015–16 [53]. These children had a mean geometric mean of 4.0 µg/L in urine and 6.2 µg of As/g creatinine, respectively. The levels of the children in this study are thus in comparison with those reported by CDC. The CDC report also presents the levels of AsIII, AsV, DMA and MMA separately. For these, it was only for DMA as a geometric mean value that could be presented separately, since the others had too many values that undercut LOD. The unadjusted geometric mean was 3.0 µg/L and adjusted, 4.3 µg As/g creatinine-adjusted in urine, respectively. In the children in this study, the creatinine-adjusted value for DMA was 4.2 µg As/g creatinine, thus on a similar level.

In a European study from 2006, comparing U-As among control and exposed children, Polish children had U-As levels of 6.0 µg As/g creatinine (girls); 6.7 µg As/g creatinine (boys) in controls and 8.0 µg As/g creatinine (girls); 8.7 µg As/g creatinine (boys) in exposed (living near a mine) [48]. Furthermore, the study found in Czech children U-As levels among control girls of 11 µg As/g creatinine and among boys 13 µg As/g creatinine, while exposed girls and boys living near a mine had AsU levels of 5.3µg As/g creatinine. A French study from 2013 among younger children (2 to 6 years old) living in naturally As contaminated areas, comparing U-As in different seasons, showed similar results with levels of 5.2 µg As/g creatinine in the summer and 5.7 µg As/g creatinine in the winter [49]. 

In the group comparison within the study sample, the only statistically significant difference was found for the content of inorganic As in urine creatinine adjusted for those who were not born/raised in the area (*p*-value: 0.049). 

### 4.4. Strengths and Weaknesses

A request to participate in the study was sent to all children in grade 2–4 (8–10 years of age) in the study area; a total of 237 children, of whom 37% (N = 87) chose to participate. As in all cross-sectional studies, there is a plausibility of selection bias. However, there is no reason to believe that the metal levels in the blood would differ between those who chose to participate in the study and those who chose not to. The results in our study are easily comparable to a previous study from the area, since the same method analytical methods of mapping heavy metals in blood were used [46,54]. For Pb and Cd, the half-life in the blood is 1.5–2 months, which in theory means that it would not affect the study population throughout the study material. Biological samples were collected during the autumn, a couple of months after the summer vacation, in order to avoid exposure from other places. The advantage of testing children is to obtain a present value that, to a lower degree, reflects individual historical exposure. Adults have a complex historical exposure via habits (smoking) and occupation, e.g., Pb is stored in the skeleton and can then be slowly excreted into the blood for decades. A limitation in the study is that true exposure for ground pollutants from the contaminated area was not known. Analyzing Cd and Pb in blood and As in urine gives the total body burden, and it is not possible to directly relate these levels to different exposure routes. There was also limited information about activity pattern, behavior for the individual children as well as pollutant levels in soil at places such as school, and residencies were the children might have stayed. This is information that could have been used to better study actual exposure from the contaminated soil. Diet is another important exposure route where only limited information were available. When levels of inorganic As and its metabolites are presented in the report, both creatinine-adjusted and unadjusted levels have been used in comparison to other studies. Creatinine adjustment was done to account for how diluted the urine was. Since creatinine has also been a biomarker for how effective the methylation of As in the body is, one could also have adjusted for the density of the urine [55]. However, creatinine-adjusted levels enabled comparison with previous studies where few density adjustments were made.

## 5. Conclusions

The results from this study show that children living and growing up in an area with highly contaminated soil do not necessarily acquire high levels of these pollutants in their bodies. These results are especially reassuring regarding Pb and Cd, since these metals accumulate in the body and have long half-lives, and are thus expected to be detected by a single blood sample. Regarding As in urine, it is more complex to capture long term exposure by a single urine sample, since the half-life is only a few days. Results from the measurements in this study do not indicate elevated levels. Thus, children in the study do not appear to be exposed to Cd, Pb and As, to such an extent that it imposes a significant increased risk of adverse chronic health effects. Even so the source of exposure, the contaminated soil, is still present in the area. Further studies, including investigating different exposure routes, are needed to better understand the exposure to children from contaminated soil. This is in order to get results that are more generalizable and can be used in actual risk assessments.

## Figures and Tables

**Table 1:** Descriptive table for the study population based on self-reported questionnaire.

Gender	N (%)
Girl	40 (46%)
Boy	47 (54%)
School	
Hovmantorp	60 (69%)
Lustigkullaskolan	14 (16%)
Björkskolan	13 (15%)
Attended grade	
2	29 (33%)
3	35 (40%)
4	23 (26%)
Born and raised in the area	
Yes	50 (58%)
No	36 (42%)
Smoking parent	
Yes	9 (10%)
No	78 (90%)
Play or cast tin soldiers, fish with Pb sinker or shoots with air rifle	
Yes	10 (12%)
No	75 (88%)
Eat locally grown vegetables	
Yes	40 (46%)
No	47 (54%)
Drink water from own well	
Yes	19 (22%)
No	67 (78%)
Eat local fresh water fish	
Yes	21 (24%)
No	66 (76%)

**Table 2:** Levels of cadmium (Cd) and lead (Pb) in blood in the study population.

Study Area			Cd (µg/L)		Pb (µg/L)	
Location	Year	N	GM	Range	GM	Range
Lessebo	2017	85	0.09	0.04–0.26	9.9	4.2–42

**Table 3:** Levels of Cd and Pb in blood for different groups in the study population. Independent t-tests have been performed between the groups to test for statistical differences.

Grouping Variable			Cd	(µg/L)		Pb	(µg/L)	
		N	GM	Range	*p*-Value	GM	Range	*p*-Value
Gender	Girl	39	0.10	0.05–0.24	0.302	10	4.6–42	0.748
	Boy	46	0.09	0.04–0.26		9.7	4.2–25	
Born and raised in the area	Yes	49	0.08	0.04–0.24	0.002	8.6	4.2–23	0.002
	No	35	0.11	0.05–0.26		12	5.9–42	
Smoking parent	Yes	8	0.08	0.04–0.19	0.359	12	5.8–42	0.443
	No	77	0.09	0.04–0.26		9.6	4.2–25	
Play or cast tin soldiers, fish with Pb sinker or shoots with air rifle	Yes	10	0.09	0.05–0.20	0.850	13	7.1–23	0.066
	No	75	0.09	0.04–0.26		9.5	4.2–42	
Eat homegrown vegetables	Yes	38	0.09	0.04–0.26	0.949	9.5	4.2–25	0.466
	No	47	0.09	0.04–0.24		10	4.6–42	
Drink water from own well	Yes	18	0.09	0.05–0.26	0.726	11	6.3–25	0.153
	No	66	0.10	0.04–0.24		9.6	4.6–42	
Eat local caught fresh water fish	Yes	21	0.09	0.42–0.24	0.693	8.7	4.6–17	0.153
	No	64	0.09	0.45–0.26		10	4.2–42	

**Table 4:** Geometric mean values for inorganic arsenic (As) with metabolites in urine unadjusted and adjusted for creatinine.

As Compound	GM (uq/L)	Range (uq/L)	GM ug/g Creatinine
DMA	4.2	0.40–44	4.8
MMA	0.47	0–4.8	0.53
AsIII	0.28	0–2.0	0.29
AsV	0.82	0.44–2.5	0.93
The sum of inorganic As	6.1	1.2–48	6.9

**Table 5:** Geometric mean values in urine for different groups of the study population. Independent t-tests have been performed between the groups to test for statistical differences.

Grouping Variable			Inorganic As	(µg/L)			Inorganic As	(µg/g Creatinine)	
		N	GM	Range	*p*-Value	N	GM	Range	*p*-Value
Gender	Girl	40	5.9	1.5–36	0.732	40	6.7	2.6–31	0.583
	Boy	47	6.3	1.2–48		46	7.2	2.5–31	
Born and raised in the area	Yes	50	5.7	1.2–48	0.367	49	6.3	2.5–31	0.049
	No	36	6.6	2.0–30		36	8.0	3.8–30	
Smoking parent	Yes	9	7.5	5.3–15	0.120	9	7.5	4.6–13	0.671
	No	78	6.0	1.2–48		77	6.9	2.5–31	
Play or cast tin soldiers, fish with Pb sinker or shoots with air rifle	Yes	10	8.8	2.3–33	0.116	10	7.4	2.9–19	0.730
	No	77	5.8	2.5–31		76	6.9	2.5–31	
Eat homegrown vegetables	Yes	40	6.4	1.7–36	0.581	40	6.8	2.5–31	0.679
	No	47	5.8	1.2–48		46	7.1	2.5–31	
Drink water from own well	Yes	19	8.0	1.8–33	0.102	19	7.2	2.6–19	0.788
	No	67	5.7	1.2–48		66	6.9	2.5–31	
Eat local caught fresh water fish	Yes	21	6.6	1.7–36	0.568	21	7.2	2.5–31	0.736
	No	66	5.9	1.2–48		65	6.9	2.5–31	

## Supplementary Materials

The following are available online at https://www.mdpi.com/1660-4601/17/20/7382/s1, File S1: Questionnaire; File S2: Consent form.
