# Supplementary material for: Cadmium and Lead Levels in Blood and Arsenic Levels in Urine among Schoolchildren Living in Contaminated Glassworks Areas, Sweden
Source: Int J Environ Res Public Health. 2020 Oct 10;17(20):7382. doi: 10.3390/ijerph17207382 (PMC7600003; doi:10.3390/ijerph17207382)
Supplement: Supplementary file 1 [file ijerph-17-07382-s001.pdf]

File S1

Questionnaire for children (To be completed together with guardians)

Date…………………

Name……………….

Personal Id-number (YYYY-MM-DD-NNNN)……………………………………………..

Gender: Girl ☐ Boy ☐

Residential address…………………………………………..

School…………………………………………………………………………………….. Class……………..

Are you born/raised in the area? Yes ☐ No ☐

If "No"; how many years have you lived here?....................................

May we take a blood sample from you? Yes ☐ No ☐

Weight: …………kg

Length: ………….cm

What does your father do for a living?...

What does your mother do for a living?...

Do you play with tin soldiers? Yes ☐ No ☐

Do you cast tin soldiers? Yes ☐ No ☐

Do you cast lead sinks? Yes ☐ No ☐

Do you shoot with air rifle? Yes ☐ No ☐

Does your mother smoke? Yes ☐ No ☐

Does your father smoke? Yes ☐ No ☐

Do you eat locally caugt fresh water fisk? Yes ☐ No ☐

If "Yes"; how many times a week?....................

Do you eat locally grown vegetables ? Yes ☐ No ☐

If "Yes"; how many times a week?....................

Do you drink water from own well? Yes ☐ No ☐

(Questionnaire translated from Swedish)

Intervjufrågor för barn (ifylles tillsammans med vårdnadshavare)

Datum: ……………………….

Namn: ………………………………………………………………………………………………….

Personnummer (ÅÅÅÅ-MM-DD-NNNN): __ __ __ __ __ __ __ __ - __ __ __ __

Kön: Flicka ☐ Pojke ☐

Bostadsadress …………………………………..

Skola ……………………………………………………………………………… Klass ………………

Är du född/uppvuxen på orten? Ja ☐ Nej ☐

Om nej; hur många år har du bott här ……………………………………..

Får vi ta ett blodprov på dig? Ja ☐ Nej ☐

Vikt: ……………… kg

Längd: …………… cm

Vad jobbar din pappa med? ……………………………………………………………………………………………………

Vad jobbar din mamma med? …………………………………………………………………………………………………

Leker du med tennsoldater? Ja ☐ Nej ☐

Gjuter du tennsoldater? Ja ☐ Nej ☐

Gjuter du blysänken? Ja ☐ Nej ☐

Skjuter du med luftgevär? Ja ☐ Nej ☐

Röker mamma hemma? Ja ☐ Nej ☐

Röker pappa hemma? Ja ☐ Nej ☐

Äter du lokalt fångad insjöfisk? Ja ☐ Nej ☐

Om ”ja”, hur många gånger i veckan? …………………….

Äter du närodlade grönsaker? Ja ☐ Nej ☐

Om ”ja”, hur många gånger i veckan? …………………….

Dricker ni vatten från egen brunn? Ja ☐ Nej ☐

(Original in Swedish)

File S2

(Translate from Swedish, original in Swedish below)

REGION KRONOBERG

To you as guardian of a children living and raised in Lessebo municipality:

Do you and your child want to help us with a blood test?

We want to investigate if there is any impact from the glassworks' pollution in primary school students (grades 2-4) born and raised in Lessebo municipality. It is a voluntary examination which means that the child leaves a small blood sample of 5 ml (a teaspoon) and a urine sample.

The study is carried out by the division of Occupational and Environmental Medicine in Lund to determine whether children who grow up in areas adjacent to old industrial properties and landfills risk having slightly elevated metal levels in the body. Since 1978, we have been carrying out corresponding measurements on children living in Landskrona and Trelleborg to investigate whether urban children have a higher metal exposure than children raised in rural areas due to for example, increased exposure from traffic and air pollution. Our studies there indicate that the surrounding environmental exposure to these substances has decreased sharply. We now want to do a similar study in your municipality to see if old industrial pollutants can have any effect on your children's blood and urine concentrations of these substances.

The project is funded by the Swedish Environmental Protection Agency's funds for health-related environmental monitoring.

What is inculded in the survey?

The blood test is performed in the most gentle way possible by nurses with great sampling habits in children. Your child gets a needle stick in the arm. During the sampling session, the child will also be allowed to submit a urine sample. We also ask you to fill in a short questionnaire about the child about leisure activities, dietary habits and about parents' smoking habits. After the sample, the child receives a compensation of SEK 100.

What do we want to measure?

We want to measure (possible) levels of lead, cadmium and mercury in blood and arsenic in the urine.

If you do not want to participate?

Your child can cancel their participation at any time. Of course, interrupting your participation has no effect on your child's care, either now or in the future.

What happens to the results?

The results and interpretation of the metal analysis will in the spring of 2018 be sent to your residential address. It is unlikely that any elevated levels of metals in the blood and urine would be drastically elevated in the children in the area due to the contaminated

soil, but if the results indicate a higher exposure, this will be communicated to the residents

in the area. If deviating test results have occurred, this will be investigated by the clinic to clarify the cause. A popular science report will be published in the autumn of 2018 in the Occupational and Environmental Medicine South report series.

(http://sodrasjukvardsregionen.se/amm/rapporter/).

The survey has been reviewed and approved by the Regional Ethics Review Board in Lund.

The blood test will be conducted on:

Kvarndammskolan

The 26th or 27th of September

We are behind the survey

Arbets- och miljömedicin SYD

Avdelningen för Arbets- och miljömedicin vid Lunds universitet

Region Kronoberg

We need your approval as a guardian. Fill in the enclosed consent form and, if your child wishes to participate, the questionnaire and send this in the attached reply envelope no later than 7 September 2017.

For questions or information, you are welcome to contact:

Kristoffer Mattisson (projektledare)

Environmental hygienist

Arbets- & Miljömedicin Syd

Skånes Universitetssjukhus

221 85 Lund

Tel: 046 - 17 72 88

e-post: kristoffer.mattisson@skane.se

Eva Assarsson

Regional Environmental nurse

Arbets- & Miljömedicin Syd

Skånes Universitetssjukhus

221 85 Lund

Tel: 046 - 222 80 22

e-post: eva.assarsson@med.lu.se

Best Regards

Kristoffer Mattisson
Arbets- & Miljömedicin Syd

Skånes Universitetssjukhus
Lunds Universitet

LUNDS UNIVERSITET
Medicinska fakulteten

Linda Vingren

Training Manager
Lessebo municipality

Conny Axelsson

Planning Manager
Lessebo municipality

Information to guardians about the processing of samples and personal data

We will store the blood samples in a biobank (Regional Biobank Register). Region Skåne and Södra Sjukvårdsregionen. Biobank Occupational and Environmental Medicine, AMMED-USIL) which is already located at the Division of Occupational and Environmental Medicine at Lund University Hospital. AMMED-USIL has a biobank license.

The samples will be stored coded. This means that they cannot be directly traced to your child as a person. The samples and the associated identification list (code key) will be stored securely and separately.

Patient data with results of the examinations (social security number, name, address, data from questionnaires and laboratory data) will be stored in a register at Occupational and Environmental Medicine. These will later be processed. Then the name and social security number will be replaced

by a code. At the time of publication of the study, no individuals can be identified. The information is protected by confidentiality. No unauthorized person has access to the information.

The samples may only be used in the way you have given your consent (we will ask you to write a consent form, if your child is to participate in the study). You have the full right to request without further explanation that your child's samples be destroyed, in accordance with the Biobanks Act.

Since the intention is to follow the development over time, the information will be preserved for the foreseeable future. Region Skåne is responsible for the handling of personal data. This is regulated by the Personal Data Act (SFS 1998: 204). If you want to know more about this law, there is detailed information at www.skane.se. By sending a signed application, you can also request information about the processing of personal data through the Personal Data Ombudsman, Region Skåne, 291 89 Kristianstad. You may also have any incorrect information corrected.

Consent to study by the metal content in the blood and urine of primary and middle school students

Students name: ……………………………………………

Personal Id-number: ……………..….-……….…….

Residential address: ………………………………………………………………………………………….

Postal code: ……………………….   City: ………………………….…………………….…

Phone number: …………………………………………………………….

Schools name:…………………………………………………….

Grade:………………………………….

I have received information regarding the study and wish to participate in the survey:

YES ☐   NO ☐

Students signature: …………………………………………………………………………………………….

Guardians signature: ………………………………………………………………………………………

Guardians signature: ………………………………………………………………………………………

(The signatures of both guardians are required if you are not the sole guardian)

Project managers underskrift: ………………………………………………………………………………

Kristoffer Mattisson

LUNDS UNIVERSITET
Medicinska fakulteten

Date:…………………………………………………

Till dig som är vårdnadshavare till barn bosatt och uppvuxet i Lessebo kommun:

Vill du och ditt barn hjälpa oss med ett blodprov?

Vi vill undersöka om det finns någon påverkan från glasbrukens föroreningar hos grundskoleelever (årskurs 2-4) **födda och uppvuxna i Lessebo kommun.**

Det är en frivillig undersökning som innebär att barnet lämnar ett litet blodprov på 5 ml (en tesked) och ett urinprov.

Undersökningen utförs av avdelningen för Arbets- och miljömedicin Syd i Lund och är till för att kunna fastställa om barn vilka växer upp i områden i anslutning till gamla industrifastigheter och deponier riskerar att ha något förhöjda metallhalter i kroppen. Vi genomför sedan 1978 motsvarande mätningar på barn boende i Landskrona och Trelleborg för att undersöka om stadsbarn har en högre metallexponering än barn uppväxta på landsbygden pga. av exempelvis ökad exponering från trafik- och luftföroreningar. Våra studier där tyder på att den omgivande miljöexponeringen för dessa ämnen har minskat kraftigt. Vi vill nu göra motsvarande studie i er kommun för att se om gamla industriföroreningar kan ha någon inverkan på era barns blod- och urinkoncentrationer av dessa ämnen.

Projektet är finansierad av Naturvårdsverkets medel för hälsorelaterad miljöövervakning.

Vad ingår i undersökningen?

Blodprovet utförs på skonsammast möjliga sätt av sjuksköterskor med stor provtagningsvana på barn. Ditt barn får ett nålstick i armen. Under provtagningstillfälle kommer barnet även få lämna urinprov. Vi ber er också fylla i en kort enkät om barnet om fritidssysselsättning, kostvanor och om föräldrars rökvanor. Efter provtagningen får barnet en ersättning på 100 kronor.

Vad vill ni mäta?

Vi vill mäta (eventuella) halter av bly, kadmium och kvicksilver i blod samt arsenik i urinen.

Om man inte vill delta?

Ditt barn kan när som helst avbryta sin medverkan. Att avbryta sin medverkan har förstås ingen påverkan av ditt barns vård, varken nu eller i framtiden.

Vad händer med resultatet?

Resultatet och tolkningen av metallanalysen kommer under våren 2018 att skickas till er bostadsadress. Det är osannolikt att eventuella förhöjda halter av metaller i blod och urin skulle vara drastiskt förhöjda hos barnen i området till följd av den förorenade marken men om resultaten indikerar på en högre exponering kommer detta att kommuniceras till de boende i området. I det fall avvikande provresultat förekommit kommer detta att utredas av kliniken för att klarlägga orsaken. En populärvetenskaplig rapport kommer under hösten 2018 publiceras i Arbets- och miljömedicin Syd rapportserie. (http://sodrasjukvardsregionen.se/amm/rapporter/).

Undersökningen är granskad och godkänd av Regionala Etikprövningsnämnden i Lund.

Blodprovet görs på:

Kvarndammskolan

den 26e eller 27e september

Vi står bakom undersökningen

Arbets- och miljömedicin SYD

Avdelningen för Arbets- och miljömedicin vid Lunds universitet

Region Kronoberg

Vi behöver ditt godkännande som vårdnadshavare. Fyll i bifogat samtyckeformulär och, om ert barn önskar delta, enkäten och skicka detta i det bifogade svarskuvertet senast den 7e september 2017.

För frågor eller upplysningar är ni välkomna att kontakta:

Kristoffer Mattisson (projektledare)

Miljöhygieniker

Arbets- & Miljömedicin Syd

Skånes Universitetssjukhus

221 85 Lund

Tel: 046 - 17 72 88

e-post: kristoffer.mattisson@skane.se

Eva Assarsson

Regionmiljösköterska

Arbets- & Miljömedicin Syd

Skånes Universitetssjukhus

221 85 Lund

Tel: 046 - 222 80 22

e-post: eva.assarsson@med.lu.se

Med vänliga hälsningar

Kristoffer Mattisson
Arbets- & Miljömedicin Syd

Skånes Universitetssjukhus
Lunds Universitet

LUNDS UNIVERSITET
Medicinska fakulteten

Linda Vingren

Utbildningschef
Lessebo kommun

Conny Axelsson

Samhällsbyggnadschef
Lessebo kommun

Information till målsmän om behandling av prover och personuppgifter

Vi kommer att lagra blodproverna i en biobank (Regionalt Biobanksregister). Region Skåne och Södra Sjukvårdsregionen. Biobank Arbets- och miljömedicin, AMMED-USIL) som redan finns vid Arbets- och miljömedicinska kliniken vid Universitetssjukhuset i Lund. AMMED-USIL har biobankstillstånd.

Proverna kommer att förvaras kodade. Det innebär att de inte direkt kan härledas till ditt barn som person. Proverna och den tillhörande identifieringslistan (kodnyckeln) kommer att förvaras på ett säkert sätt och åtskilda.

Patientuppgifter med resultat av undersökningarna (personnummer, namn, adress, uppgifter från frågeformulär och laboratoriedata) kommer att lagras i ett register vid Arbets- och miljömedicin. Dessa kommer senare att databehandlas. Då kommer namn och personnummer att ersättas av en kod. Vid publikation av studien kan inga enskilda individer identifieras. Uppgifterna är skyddade av sekretess. Ingen obehörig har tillgång till uppgifterna.

Proverna får endast användas på det sätt som du har gett samtycke till (vi kommer att be dig skriva på en samtyckesblankett, ifall ditt barn ska delta i studien). Du har full rätt att utan närmare förklaring begära att ditt barns prover ska förstöras, i enlighet med lagen om biobanker.

Eftersom avsikten är att följa utvecklingen över tid kommer uppgifterna att bevaras under överskådlig tid. Region Skåne är ansvarig för hanteringen av personuppgifterna. Detta regleras av Personuppgiftslagen (SFS 1998:204). Vill du veta mera om denna lag finns utförlig information på www.skane.se. Genom att skicka en undertecknad ansökan kan du också be att få uppgifter om personuppgiftsbehandlingen genom Personuppgiftsombudet, Region Skåne, 291 89 Kristianstad. Du kan också få eventuellt felaktiga uppgifter rättade.

Samtycke till undersökning av metallhalten i blod och urin hos låg- och mellanstadieelever

Elevens namn: …………………………………………

Personnr: ……………..…-…………….

Bostadsadress: ……………………………………………………………………………………….

Postnummer: ………………………. Ort: ……………………………..……………………

Telefon: …………………………………………………………….

Skolans namn:……………………………………………………

Klass:………………………………….

Jag har tagit del av information rörande studien och önskar delta i undersökningen:

JA ☐ NEJ ☐

Elevens underskrift: …………………………………………………………………………………………

Målsmans underskrift: ………………………………………………………………………………………

Målsmans underskrift: ………………………………………………………………………………………………

(Båda vårdnadshavares namnteckning behövs om man inte är ensam vårdnadshavare)

Projektledarens underskrift: ………………………………………………………………………………

Kristoffer Mattisson

Datum:…………………………………………………

LUNDS UNIVERSITET
Medicinska fakulteten
